# P061: Early detection and successful control of vancomycin resistant Enterococcus faecium (VRE) outbreak in an academic hospital in Pretoria, South Africa

**DOI:** 10.1186/2047-2994-2-S1-P61

**Published:** 2013-06-20

**Authors:** RM Lekalakala, E Iewis, E Silberbauer

**Affiliations:** 1 Medical Microbiology, NHLS & University of Pretoria, Midrand, South Africa; 2 IPC Division Steve Biko Academic Hospital, South Africa; 3 NHLS & University of Pretoria, Pretoria, South Africa

## Introduction

Active surveillance which was introduced in 2008 following the Clostridium difficile outbreak in our hospital enhanced early detection of vancomycin resistant *Enterococcus faecium*outbreak in the medical intensive care unit (MICU). Prompt intervention strategies which were implemented by infection control team facilitated the successful control.

## Background

A maximum of 10 VRE isolates per year has been the norm between 2008 and 2011 in our 800 bed hospital. These isolates were from the oncology units. Between the 4^th^ and 30^th^ August 2012, 7 patients in MICU had VRE positive cultures from a variety of samples including blood cultures.

## Methods

Sample types and patient clinical data were collected; movement of infected patients within the hospital was traced to identify the possible index patient for patients who were not admitted directly into MICU. Hand washing and contact precautions practises were audited and reinforced as per VRE policy. Environmental cleaning audits were done routinely to minimize the bio burden and the unit was closed for new admission other than VRE positive patients. Infection control monthly reports were reviewed from May 2012 for possible oversight as there were no VRE cases in April. Monitoring and recording of new case is on-going with daily update to the team. One Isolate per patient is stored for molecular characterization.

## Results

28 patients were identified over 10 months (May 2012 to February 2013). 42% (12/28) patients from MICU, 25% (7/28) were dialysed. The environmental cleaning audit revealed a leaking sewage pipe in the MICU which resulted in a complete closure of the unit and immediate repairs which was followed by terminal cleaning of the whole unit.

## Conclusion

It is well documented the most transmission of VRE is via contaminated hands of health care workers or environment or patient equipments. The findings in this study suggest that environmental contamination was the source of the VRE.

## Disclosure of interest

None declared

